# Coaching in Competence by Design: A New Model of Coaching in the Moment and Coaching Over Time to Support Large Scale Implementation

**DOI:** 10.5334/pme.959

**Published:** 2024-02-06

**Authors:** Denyse Richardson, Jeffrey M. Landreville, Jessica Trier, Warren J. Cheung, Farhan Bhanji, Andrew K. Hall, Jason R. Frank, Anna Oswald

**Affiliations:** 1 Department of Physical Medicine and Rehabilitation, Queen’s University, Kingston, ON, Canada; 2 Royal College of Physicians and Surgeons of Canada, Ottawa, ON, Canada; 3 Department of Emergency Medicine, University of Ottawa, Ottawa, ON, Canada; 4 Education, Faculty of Medicine and Health Sciences, McGill University, Montreal, QC, Canada; 5 University of Ottawa Faculty of Medicine, Ottawa, ON, Canada; 6 Division of Rheumatology, Department of Medicine, Faculty of Medicine and Dentistry, University of Alberta, Edmonton, ON, Canada; 7 Competency Based Medical Education, University of Alberta, Edmonton, AB, Canada

## Abstract

Coaching is an increasingly popular means to provide individualized, learner-centered, developmental guidance to trainees in competency based medical education (CBME) curricula. Aligned with CBME’s core components, coaching can assist in leveraging the full potential of this educational approach. With its focus on growth and improvement, coaching helps trainees develop clinical acumen and self-regulated learning skills. Developing a shared mental model for coaching in the medical education context is crucial to facilitate integration and subsequent evaluation of success. This paper describes the Royal College of Physicians and Surgeons of Canada’s coaching model, one that is theory based, evidence informed, principle driven and iteratively and developed by a multidisciplinary team. The coaching model was specifically designed, fit for purpose to the postgraduate medical education (PGME) context and implemented as part of Competence by Design (CBD), a new competency based PGME program. This coaching model differentiates two coaching roles, which reflect different contexts in which postgraduate trainees learn and develop skills. Both roles are supported by the RX-OCR process: developing **R**elationship/**R**apport, setting e**X**pectations, **O**bserving, a **C**oaching conversation, and **R**ecording/**R**eflecting. The CBD Coaching Model and its associated RX-OCR faculty development tool support the implementation of coaching in CBME. Coaching in the moment and coaching over time offer important mechanisms by which CBD brings value to trainees. For sustained change to occur and for learners and coaches to experience the model’s intended benefits, ongoing professional development efforts are needed. Early post implementation reflections and lessons learned are provided.

## Introduction

New teaching and learning practices across the continuum of medical education are required to fulfill the promise of competency based medical education (CBME) as learner-centered, developmental education. Competency-focused instruction is a core component of CBME that facilitates the developmental acquisition of competencies [1], and coaching is a salient avenue to accomplish this. The trajectory of development will vary among learners. Therefore, instruction and guidance must be tailored to each individual’s existing level of ability and their learning goals to enable the full benefit of competency-focused instruction. Coaching focuses on progressive improvement and utilizes individualized guidance to promote growth and development [2,3], aligning with competency-focused instruction, while also attending to the need for individualized guidance. Increasingly, coaching is emerging as an approach to assist individual medical learners in their pursuit of progressive competence and professional development [4,5]. As such, coaching is critical in realizing the full potential of CBME [1].

Coaching is one of many approaches that can be utilized to provide guidance to learners across the continuum of medical education. With the growing interest in coaching in medical education, clarifying its purpose and differentiating it from mentoring and teaching is important [6,7]. While it can be argued that mentoring, teaching, and coaching all focus on an individual’s development, an increasingly prevalent distinction is the coach’s orientation toward asking over telling; a coach asks powerful questions to catalyze learner self-reflection, leading the learner in self-discovery, whereas a mentor, advisor, or teacher imparts information or advice based on their own past experience or expertise. Additionally, coaches are improvement-oriented (for future practice) rather than problem-oriented. [8,9]. Competency frameworks provide the necessary clarity on the goals of training, while a coach supports and facilitates the learner in their understanding of their individual trajectory to achieve those goals. Coaching focuses on future performance: the coach guides the learner through a process of reflection, which enables them to identify personalized goals within the larger framework and create individualized pathways for their learning [4,6]. By doing so, the coach is promoting autonomy in the learner that contributes to their intrinsic motivation to continue to grow and develop, while also facilitating self-regulated learning skills [10]. Coming to this shared understanding of the purpose of coaching will promote consistent implementation, useful faculty development supports and program evaluation of its impact.

Feedback is also an important means of improving learner performance [11,12]. Yet, despite decades of effort, it continues to be fraught with challenges, from both the learner and teacher perspectives and hence has fallen short of its potential. [13,14,15,16,17,18,19,20,21,22]. In 1983, when Ende introduced feedback into medical education, he referred to feedback as “information describing students’ or house officers’ performance in a given activity that is intended to guide their future performance in that same or in a related activity [11].” Feedback provides information about what has just happened but, all too often is received by learners well after the fact, asynchronously and in written form, without the important learning conversation that brings practical meaning to the words. That said, feedback in many cases will occur as part of, or even before coaching but, coaching assures that the learner and coach co-create a realistic actionable plan for future improvement in a relevant practice area. Interestingly, in the last decade, in attempts to address some of the challenges identified with feedback, the scholarly discourse related to feedback has shifted, conceptually, calling attention to the vital need to engage the learner in a reflective conversation resulting in actionable steps for improvement as well as attending to the important relational and sociocultural factors influencing the utility of feedback [23,24,25,26]. This evolving conceptualization of these new pragmatics of feedback include basic essential elements in a coaching relationship.

While coaching may still be considered an emerging approach in medical education, it is by no means a new concept in other domains. The word “coach” dates back to the mid-16^th^ century. Its present-day meaning, referring to a person who guides or facilitates learning toward an end goal, originated in the academic context at Oxford University in 1830 [2]. As a means of promoting development, coaching has expanded in multiple high-performing professional domains, (e.g., sports, the arts, business) over the last 50 years [27,28,29,30,31]. Despite the prevalence of coaching, a unified approach does not exist. The diversity related to the many different coaching approaches, having drawn from a wide range of knowledge bases and applied in such variable contexts [32] highlights the crucial imperative of having a coaching model that is matched to the uniqueness of a context in which it will be used.

The distinctive contexts of medical learning, together with the complexity of the sociocultural interaction involved in coaching, necessitate a new coaching model for CBME that provides a shared understanding of the purpose of coaching and the coaching roles and associated processes required. In this paper, we describe this new fit for purpose coaching model developed to support large scale CBME implementation, and reflect upon lessons learned through this process. The model addresses gaps in the current literature, including application of coaching in the moment, in clinical environments juxtaposed closely in time to an observed clinical activity; emphasizing the need for observation in the clinical environments; but, also the need for a bidirectional coaching learning conversation; encouraging learners to truly reflect and together with their coach, to co-create individualized learning goals aimed at improved future practice habits. The inclusion of coaching conversations and processes in the CBD model allows this CBME model to be more than a shift in assessment practices and regulations. It encourages a genuine change to embrace a learning culture, emphasizing the wealth of learning opportunities within the medical education environment. Coaching in the moment facilitates the improved clinical acumen and practice while in addition to that, coaching over time also supports the developmental arcs of the learners as well as the professional identity formation of them as physicians and lifelong learners.

This CBD coaching model, with resident learning squarely as the primary focus, including its two distinct yet complementary coaching roles, Coaching in the Moment (CiM) and Coaching over Time (CoT), as well as the associated process tool, RX-OCR not only provides a shared mental model to ensure the fidelity of implementation of coaching (i.e., to ensure that training programs employ the key components of the model after implementation) but also allows for the much-needed iterative evaluation of the coaching process, for the coach, the learners, and the overall coaching program.

## The Royal College of Physicians and Surgeons of Canada coaching model

**Figure 1:**
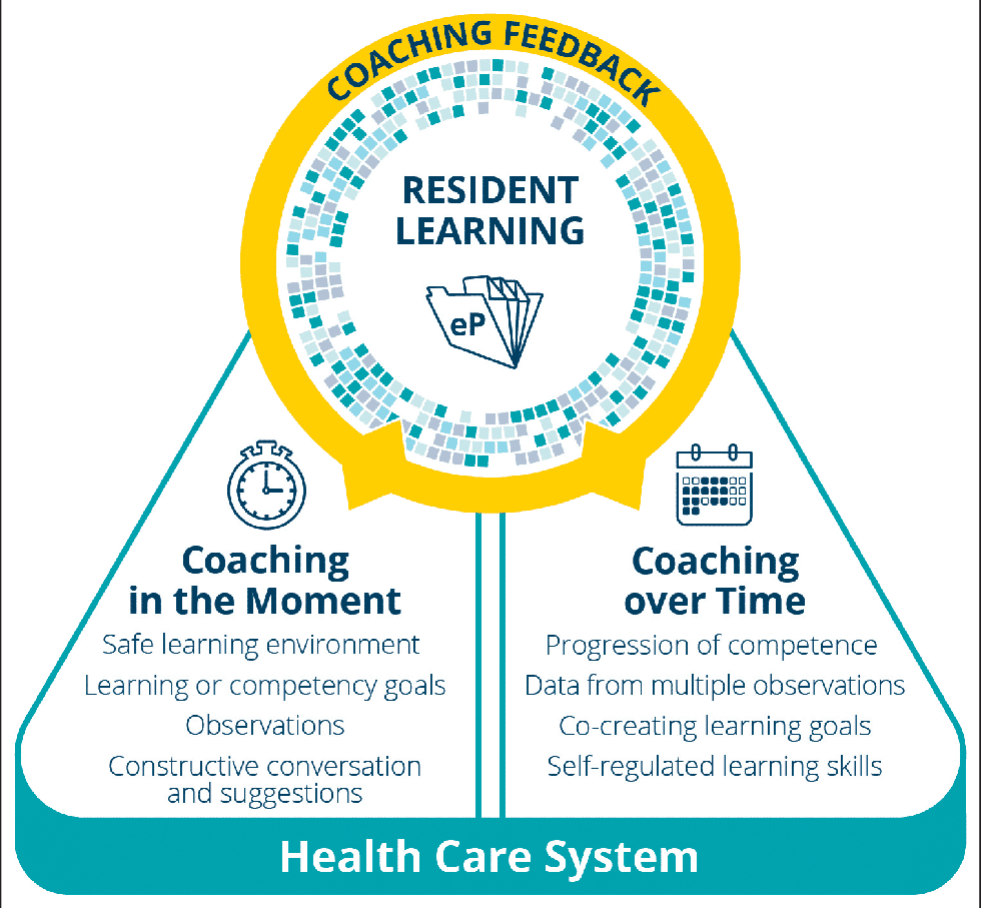
The Competence by Design (CBD) Coaching Model. Resident learning is the focal point, reflecting the importance of a learner-centered, developmental approach to competency acquisition. Two distinct coaching roles support that development: coaching in the moment and coaching over time. The graphic also illustrates other important notions that are fundamental to the Competence by Design (CBD) Coaching Model. First, resident development is captured by documentation that is kept in a learning portfolio, which is readily available for self-reflection and guided reflection activities. The pixelated ring (blue and gray) is a graphical representation of the increasingly more complete depiction of the resident’s competence as development of expertise occurs. There is a bidirectional connection (yellow arrows) between the resident and the coach for both coaching in the moment and coaching over time, illustrating the importance of the collaboration and relationship, an “educational alliance,” [23] that must be nurtured, for the benefit of resident learning and development. Finally, the CBD Coaching Model also explicitly recognizes that resident learning and coaching are, for the most part, embedded in the complex workplace of the Canadian health care system, which presents multiple competing priorities for all clinicians, the most significant of which is the paramount importance of delivering safe, high-quality care to patients [36].

The Royal College of Physicians and Surgeons of Canada (Royal College) introduced a major change initiative to reform postgraduate specialist education in Canada, [33] called Competence by Design (CBD), in keeping with the global movement of CBME [34]. Coaching was identified as an essential educational component of this initiative [1,35] and the CBD Coaching (to Competence) Model was developed. The CBD Coaching Model (see Figure 1) is a theory based, evidence informed, principle driven model that was iteratively developed by a multidisciplinary team with expertise in coaching and medical education domains. [33,36] This coaching model was specifically created, fit for purpose to the PGME context for the explicit purpose of supporting and facilitating each trainee’s individual learning and progressive development of competence. The model emphasizes the importance of a learner-centered, developmental approach to competence, since coaching facilitates progressive development via individualized instruction and guidance tailored to each unique learner’s stage of competence along the trajectory of development. Integral to the model, the associated process tool, RX-OCR, was created to emphasize the importance of the crucial elements of a coaching relationship and process. The CBD model importantly includes the role of a Coach in the Moment (CiM), which speaks to the pragmatic coaching role in clinical settings. CiM is not necessarily requiring a longitudinal coaching relationship. Both these two specific elements are not addressed by existing coaching models or descriptions in the medical education literature. The coaching process, RX- OCR, highlights the importance of rapport or relationship building as well as emphasizes the need for an explicit articulation of the expectations of the coaching process. However, the CBD model also emphasizes the value of this occurring in advance of but, also iteratively being revisited, as demarcated by the hyphen, before observation, directly or indirectly, of clinical work. Additionally, an explicit emphasis on observation as the cornerstone for coaching sets this CBD model apart from others. Lastly, with learning and improvement as the ultimate goal, the CBD Coaching Model underscores the importance of the coaching conversation co-identifying actionable steps to promote growth and development in a psychologically safe environments and in settings, both clinical (coaching in the moment) and non-clinical (coaching over time). The aim of the overall process and coaching itself is to optimize the likelihood that the learner will receive and incorporate the guided reflections and make improvements for their future practice.

The CBD Coaching Model was purposefully designed with two distinct coaching roles: 1) coaching in the moment (CiM) and 2) coaching over time (CoT). These roles have unique functions, while both support trainees’ progressive development. Importantly, both roles value observed work in the clinical environment as learning opportunities. They also both place the learner’s needs at the forefront. The duration of the coaching conversation, the goals attended to, the content addressed, and even the context in which the conversation takes place will differ between the roles. Any clinical teacher, who interacts with learners in the clinical environment would use CiM, whereas a smaller number of specifically designated faculty coaches would apply CoT. Together, these coaching roles support trainees’ professional development in unique but complementary ways.

### Coaching in the moment

CiM occurs in the clinical environment between a clinical teacher and trainee, at or close to the moment in time that the trainee is observed engaging in a clinical activity. CiM demands that the long-established role of the clinical teacher evolve from the traditional supervisory role focused on quality of care to one that is also focused on facilitating that learner’s growth and continual improvement as they progress through their formal training. Observations done as part of CiM are intended to be low-stakes observations of authentic daily work; single observations are not used for any progression decision on their own; rather, together many observations form an aggregate of data points in the trainee’s learning portfolio, upon which the learner can reflect [33,37]. CiM utilizes these individual observations as the key foundational ingredient to underpin brief “in the moment” coaching conversations to facilitate individual trainee development. Given the busy clinical environment in which CiM conversations take place, it is expected that these conversations will be brief, in most cases only a few minutes long. The desired outcome of the coaching, often by co-creation, is that the trainee understands specific improvement(s) or goals for improvement in future similar clinical encounters. In this way, the coach is facilitating the trainee’s development from their current place toward a desired future competence, but in a graduated fashion. The full potential of CiM, therefore, will only be realized if it becomes a normal, expected, and embedded daily educational activity in all clinical learning settings.

### Coaching over time

CoT requires a longitudinal relationship between a designated coach and trainee. The CBD Coaching Model explicitly assigns CoT a dual purpose: it concurrently focuses on helping the trainee to become both an independent, competent clinician and one who is prepared for a career with continued competence as a self-regulated learner. Each CoT relationship is intended to last longer than any one clinical experience, but the specific duration is not currently delineated. Therefore, in some programs, a CoT alliance may span the entire postgraduate training period whereas in other programs, trainees may have a series of sequential CoT relationships, each not less than several months’ duration. In all CoT relationships, regularly scheduled face-to-face discussions about the trainee’s progression toward competence are essential and they should occur across the entire duration of the postgraduate training period. Unlike the observations that inform CiM, the observations that inform CoT encounters are made by reviewing and reflecting on the data collected in the trainee learning portfolio as well as noting behaviours during CoT coaching meetings, but importantly, they also include reflections that the trainee has made about their own performance or professional development. On the basis of any of these types of observations, the CoT coach will guide, often by co-construction in early stages, the trainee to establish learning goals and develop a clear action plan to improve their performance. For this type of relationship to develop and work well, trainees must feel confident that their coach has their best learning interests in mind [23].

## Coaching is a Process: RX- OCR, a process tool supporting both the role of Coaching in the Moment and Coaching over Time

The structure of the CBD Coaching Model, with both CiM and CoT encouraging 1:1 coaching conversations, underscores a key principle of coaching: the provision of individualized guidance to facilitate learning and progressive development. However, a 1:1 coaching conversation for improvement is only one part, albeit an important one, of a larger process that is needed for effective coaching to occur. To facilitate effective CiM and CoT, coaches are guided by a step-by-step process denoted by the acronym RX-OCR in the CBD Coaching Model (see Table 1). RX-OCR is an integral process tool of the model, which is intended to support practical implementation and enable the application of embedded key coaching principles. Use of the RX-OCR process promotes coaching irrespective of the duration of the clinical experience and provides all coaches with a guiding framework for preparing for and engaging in coaching.

**Table 1:** The RX-OCR process in the CBD Coaching Model.


**Relationship/Rapport**	Establish an educational **R**elationship/**R**apport between the trainee and the coach (“educational alliance”) to establish a safe learning environment

**Expectations**	Set e**X**pectations for an encounter (discuss learning goals)

**Observe**	**O**bserve the trainee and the trainee’s work (directly or indirectly)

**Coach**	**C**oach the trainee for the purpose of improving that work

**Record/Reflect**	**R**ecord a summary of the coaching encounter and **R**eflect


The RX-OCR process has five key steps and is designed to be an iterative process. The remainder of this section will describe each step and highlight the underlying coaching principles.

### Steps 1 and 2: Establish a Relationship/Rapport and set eXpectations, *RX-*

The first two steps, RX, are believed to be critical for coaching to be successful [23,24]. Given that the primary purpose of coaching is to facilitate growth and development, coaching practices that support trainees in adopting a growth mindset are vital [38]. By starting with and separating the RX steps in the RX-OCR coaching process, this tool emphasizes that the coach’s work in establishing a relationship with the trainee, building rapport, and ensuring clear expectations are foundational to coaching [25,26,27,28,29,30,31,32,33,34,35,36,37,38,39]. Importantly, this includes creating a safe learning environment. Psychological safety in learning environments is imperative to allow trainees to take interpersonal risks for the sake of their learning and development without fear of repercussion, and to embrace a growth mindset [40,41,42,43,44]. The importance of establishing this safety cannot be overstated: it is a fundamental principle of coaching [45,46]. When a coach articulates to the trainee their commitment to the individual’s development, the coaching relationship is strengthened and the message that the focus is on development and not on judgment is reinforced [23,47].

The X in RX highlights the importance of ensuring that expectations are clearly articulated and mutually understood by both the coach and trainee, including those related to the overall coaching process and those associated with any specific coaching encounter. To ensure clarity of expectations and to ground coaching in an individual’s needs, trainees must be actively engaged in setting goals before any coaching. This key principle empowers trainees to be active partners in their learning and development [48]. When successful, the RX steps should help trainees shift away from a fixed mindset toward a growth mindset and set the stage for impactful coaching conversations.

The hyphen within the RX-OCR acronym is intended to emphasize these initial foundational steps and remind coaches to revisit the first two steps of the process at the beginning of each coaching encounter. It also acknowledges, particularly in CiM, that these first two steps do not always need to immediately precede the observation of work but should be done in close proximity.

### Step 3: Observe, *O*

Observation of trainees’ work is the key ingredient in both CiM and CoT. Observation may occur directly or indirectly. Direct observation refers to the process of watching trainees perform a task to develop an understanding of how they apply their knowledge and skills to practice [49,50]. Indirect observations are ones that an individual makes without having directly watched the trainee perform the task. Indirect observation can include gathering information from surrogate data, such as a trainee’s oral case presentation, clinical documentation, reports from other health care providers, patients, or families, or from the trainee’s learning portfolio. In the CoT role, some coaches find it easier to conceptualize the indirect observation (or reviewing) of available data as obtaining information. That said, there are opportunities for CoT coaches to directly observe a trainee’s self-regulated learning skills.

Observation is done primarily for development and improvement (formative or “for learning”) [51]. Observation allows the coaching to be relevant to where the trainee is situated on their own individualized developmental trajectory. Frequent low-stakes observations create authentic snapshots of competence that converge and aggregate to give a more fulsome representation of a trainee’s competence. It is only once multiple observations, in a variety of clinical circumstances, observed by multiple coaches, have occurred that competence committees make judgments on trainee competence based on the aggregate of data and the trajectory of development [52].

### Steps 4 and 5: Coach, *C*, Record/Reflect, *R*

Following an observation, the trainee and coach should engage in a coaching conversation. The coach’s approach to the coaching conversation is context specific (CiM or CoT), and the conversation may take different forms depending on the preferences of the trainee and coach. Using a reflection-based approach, where the coach promotes reflection that leads the trainee to self-identify what is required for improvement, would further encourage the trainee to be an active partner in their learning. However, in some contexts, a directive or “autocratic” approach is more effective [8,53], often utilized in sport and music, whereby the coach provides specific suggestions for improvement. Regardless of what type of coaching conversation occurs, the trainee should clearly co-construct the specific steps for improvement with their coach.

The final, but equally important step, is to record a summary of the coaching encounter (R), including the learning and improvement points, and reflect. Once recorded, the coaching encounter becomes a part of the trainee’s learning portfolio, which facilitates future reflection by the trainee, upon which improvement can be based. Although not every coaching conversation needs to be recorded, in a learning culture that ensures a safe learning environment and embraces a growth mindset, the benefits of coaching will be maximized when it is done frequently to provide iterative “nudges” rather than infrequent larger corrections. Recording the outcomes of coaching encounters is also part of programmatic assessment in CBME [54]. Competence committees rely on these records, in aggregate, to judge trainee competence and then make recommendations about trainee progression and promotion within a training program.

Finally, both trainees and coaches are encouraged to regularly reflect on their coaching encounters. Reflection is a fundamental coaching principle that underpins the process of improvement and its use is encouraged by the structure of the CBD Coaching Model and the RX-OCR tool. Trainees are encouraged to initially self-reflect and then discuss their reflections with their CoT coach. Coaches are also encouraged to reflect on ways they can improve future coaching encounters.

## Reflections and lessons learned

The introduction of coaching as part of the large-scale national implementation of a transformative change in postgraduate medical education has, not unexpectedly, had some challenges. In this final section, we discuss some of these challenges, to provide insights and lessons learned to assist others who might undertake similar changes.

### Challenge 1 – It can be difficult to incorporate direct observation and coaching into busy clinical practice

While direct observation is key to effective coaching, clinicians face many competing priorities and pressures in busy clinical practice environments [55,56]. Even though a coach need not observe a whole patient assessment, the observation and the associated coaching still take time.

Many coaches have found it easier to conduct observations if they employ strategies such as dedicating a specific time to direct observation within their clinical workflow (eg., conducting observations with the first or last patient or case of the day, or one or two inpatients with clinical findings to review during rounds) or asking the trainee to demonstrate a specific part of the patient assessment after the case review [50]. Indirect observations, which can occur in many ways (e.g., oral case review, workplace documentation review, discussions with other health care professionals) can also be used as the basis for coaching interactions, but should be in addition to some direct observation. The process of implementing CBD revealed that supervisors and trainees need to be intentional about making time for coaching, soon after the observation. Planning ahead is crucial to ensure both occur.

### Challenge 2 – Physicians are trained to solve problems and coaching requires a new skill set

Physicians have traditionally been socialized and trained to solve problems and address issues; the inquiry approach in coaching can be foreign to them. The natural tendency for a physician acting as a coach in CiM or CoT is to propose solutions to an identified gap as opposed to guiding the learner to find the solutions. Also, coaching requires skills that have not traditionally been part of medical education curricula and therefore are not part of most clinicians’ skill set.

Orienting trainees and faculty to the inquiry approach will probably help, but targeted professional development is also needed. The Royal College offers resources on both CiM and CoT to assist with faculty development (https://www.royalcollege.ca/rcsite/cbd/implementation/wbas/coaching-and-cbd-e), but local applied practice is also important. Emerging evidence suggests that co-learning (trainees and faculty together) about coaching can be advantageous [57]. Specific to CoT, having the trainee complete a self-reflection tool before meeting with their coach may expedite adoption of the inquiry approach. This shift will take time.

### Challenge 3 – Learners perceive observation as summative assessment related to the prevailing performance-oriented culture

In CBD, observed work serves a dual purpose. Observations form the basis for coaching but are also recorded in the trainee’s learning portfolio, and reviewed as an aggregated whole as described in a programmatic assessment model for competence committees. This dual purposing has contributed to the learners’ perception that any observed work is a summative assessment [58,59]. A learner’s experience is entangled in the culture in which they are learning [60]. Not surprisingly, then, the still-prominent assessment-focused performance culture of medical education promotes this view [58,59].

This challenge underscores the importance of developing a shared mental model and ensuring transparency in the coaching process. Observations should be framed as workplace-based learning rather than workplace-based assessment (WBA), highlighting behaviours and practices that support trainees’ adoption of a growth mindset in clinical settings. It is crucial to develop coaching relationships that ensure a psychologically safe learning environment, establish mutually understood goals and expectations, and support the desired improvement-oriented culture [61]. One important way to maintain psychological safety is a shared understanding of what the recorded observations are going to be used for, and with whom the information is going to be shared. What cannot be understated however, is the influence of our predominant current judgement, performance-focused culture on the learner’s perception of the need to perform. The primary goal should be to assure that all, learners, faculty, medical education organizations and systems are focused on the shared purpose of developing individuals into the best physicians they can be to serve the Canadian population in the continuum of care for health. To do so, the systems must foster a safe learning environment and provide the necessary supports for learners and faculty to embrace a learning culture. The second R in the RX-OCR process was initially proposed to stand for Record alone; however, the recorded observations permit trainees to self-Reflect on their past performance to co-create learning goals with their coach, in keeping with a growth orientation.

## Conclusion

Coaching is a complex human interaction that involves multiple factors at the individual, relational, and systems levels, all of which require attention. The CBD Coaching Model and its associated implementation tool, RX-OCR, developed as part of the Royal College CBME change initiative, have been described, highlighting the key principles of coaching that guided their design. Successful implementation of the CBD Coaching Model in the unique context of medical education will take time and require sustained effort, especially considering that the approach is a novel one in medical education. Reflections and lessons learned from implementation have been shared to inform others who are contemplating or already working in this area. While implementation of CBME approaches requires an investment of resources, emerging evidence suggests that the benefits of coaching may be a return on that investment, particularly for learners but also for coaches.

## Disclaimer

The views and opinions expressed in this article are those of the authors and do not necessarily reflect the official policy or position of the Royal College of Physicians and Surgeons of Canada (“Royal College”). Information in this article about Competence by Design (“CBD”), its implementation and related policies and procedures do not necessarily reflect the current standards, policies and practices of the Royal College. Please refer to the Royal College website for current information.
